# Open Source 3D Multipurpose Measurement System with Submillimetre Fidelity and First Application in Magnetic Resonance

**DOI:** 10.1038/s41598-017-13824-z

**Published:** 2017-10-18

**Authors:** Haopeng Han, Raphael Moritz, Eva Oberacker, Helmar Waiczies, Thoralf Niendorf, Lukas Winter

**Affiliations:** 1 Berlin Ultrahigh Field Facility (B.U.F.F.), Max-Delbrück Center for Molecular Medicine in the Helmholtz Association, Berlin, Germany; 2 Experimental and Clinical Research Center (ECRC), a joint cooperation between the Charité and the Max Delbrück Center for Molecular Medicine, Berlin, Germany; 3 MRI.TOOLS GmbH, Berlin, Germany

## Abstract

Magnetic resonance imaging (MRI) is the mainstay of diagnostic imaging, a versatile instrument for clinical science and the subject of intense research interest. Advancing clinical science, research and technology of MRI requires high fidelity measurements in quantity, location and time of the given physical property. To meet this goal a broad spectrum of commercial measurement systems has been made available. These instruments frequently share in common that they are costly and typically employ closed proprietary hardware and software. This shortcoming makes any adjustment for a specified application difficult if not prohibitive. Recognizing this limitation this work presents *COSI Measure*, an automated open source measurement system that provides submillimetre resolution, robust configuration and a large working volume to support a versatile range of applications. The submillimetre fidelity and reproducibility/backlash performance were evaluated experimentally. Magnetic field mapping of a single ring Halbach magnet, a 3.0 T and a 7.0 T MR scanner as well as temperature mapping of a radio frequency coil were successfully conducted. Due to its open source nature and versatile construction, the system can be easily modified for other applications. In a resource limited research setting, *COSI Measure* makes efficient use of laboratory space, financial resources and collaborative efforts.

## Introduction

Magnetic resonance imaging (MRI) is a vital clinical tool for diagnosis and for guiding therapy, which has been described as the single most important medical innovation^1,2^. MRI is a versatile instrument for clinical science and the subject of intense research interest. Research directions focus on conventional and emerging applications^3–17^ facilitated by enabling technology including new magnet design^18–20^, gradient coil developments^21,22^, novel radio frequency technology^23–29^, techniques for probing and shimming magnetic fields^30,31^, transmission field mapping and shaping^32,33^, electric property tomography^34^, thermal interventions and RF heating^35–37^, magnetic resonance safety and MR compatibility^38–42^. Advancing this research requires high fidelity measurements in quantity and location of the given physical property, including E-fields, H-fields and temperature.

To meet this need a portfolio of instruments supporting temperature, E-field or H-field probes are commercially available. The ubiquity of and enthusiasm for these instruments is strongly hampered because they are costly and commonly customized if not limited to a specified application. Mapping a tiny volume might be sufficient for E- or H-field measurements of small and mid-size RF antennae^43^. Yet, examining the characteristics of large volume or whole body RF antennae requires enhanced coverage including the whole magnet bore. The spatial resolution requirements for E- or H-field measurements in test objects might be sufficient within a centimetre resolution. MR safety applications like RF induced E-field or temperature measurements of subtle passive conductive medical implants might require sub-millimetre resolution though^39,44^. The performance of RF coils or gradient coil inserts can be evaluated outside of the MR magnet. Yet, main magnetic field characterization or measurements during MRI scans might require sensors being positioned and moved inside the MR magnet. Supporting a broad range of applications requires the capability to position the measurement device horizontally and vertically.

Developing a measurement system that meets the requirements of spatial fidelity without the use of any magnetic components constitutes a challenge, is costly and might come with the caveat of offsetting if not losing its versatility and capability to support a broad range of applications. Common 3D printers or XYZ tables can be potentially used in conjunction with E-field and H-field probes to map the field of RF coils, however they are not strong enough to facilitate long probes that reach into the MR scanner bore, are limited by the mapping volume or lack an open source nature in order to adjust the system for the intended application^41,43,45^.

Recognizing these limitations this work presents an automated open source measurement system that provides submillimetre resolution for a material cost of ~2000€. The development of this measurement system is part of the cost effective open source imaging (COSI) initiative aiming to build an affordable open source MR system and dedicated research hardware^46–48^. *COSI Measure* can be used together with various probes including electromagnetic, temperature and ultrasound sensors in magnetic resonance research and in related fields. Due to its open source nature and versatile construction, the system can be easily modified for other applications such as computer numeric control machinery, 3D printing, surface-mount device soldering etc. The open source nature of *COSI Measure* (updates will be posted on www.opensourceimaging.org) allows the research community to share and enjoy improvements, upgrades and modifications of the system, which simplifies and accelerates the reproducibility of research results. In a resource limited research setting, *COSI Measure* makes efficient use of laboratory space, financial resources and collaborative efforts.

## Methods

### Mechanical subsystem

The mechanical subsystem has been designed in a 3D CAD (computer-aided design) model using SketchUp Make (version 15.3.331, Trimble Navigation Limited, Sunnyvale, CA, USA). It is designed as a three axis linear stage^49–51^, with a robust aluminium based frame^52^. A strong and robust setup was intended to accommodate a broad spectrum of measurement, construction or scientific tools such as electromagnetic field probes, temperature sensors, drills, plotters, saws, pipettors etc. The base frame is made of 11 aluminium profiles and has the dimensions of (880 × 960 × 840) mm³, so that it is able to pass through a door (standard width = 940 mm). Three ball screws (one for each axis) which consist of 5 mm threaded shafts and ball bearings have been used to translate rotational motion to linear motion. A second ball screw can be mounted along the x-axis for applications that need higher forces (e.g. CNC applications). The advantages of using ball screws are high precision and good reproducibility, which are key features for measuring physical parameters. We use linear bearings and supported rails to support the mechanical movement for the x- and y-axis while precision shafts and linear bearings are used to guide the movement along the z-axis. Ball screws and floated bearings are mounted on customized aluminium (10 mm thickness) plates. Limit-switch- and cable-chain-holders were designed in 3D CAD and 3D printed with ABS (acrylonitrile butadiene styrene) material. A movable lift table allows mobility of the system and provides the possibility of adjusting the height of the machine.

Assembly instructions, 3D CAD models of the complete system, the aluminium plates and limit switch holders, as well as the numbered part list with cost, quantity and vendor are available in the files provided, with an overview given in Table 1.

**Table 1:** Design files for the mechanical subsystem.

File/folder	Contents
assembly_instructions.docx	This file describes the assembly process of the machine step by step.
Aluplates	This folder contains all the SketchUp design files for the aluminum plates. Files in this folder are named in the format of Pn_XxYxZ, with Pn being the name of the plate, X, Y and Z being the dimensions of the plate.
Limit Switch holder	This folder contains all the design files for the limit switch and cable chain holders.
complete_system.skp	This file is the SketchUp design for the whole system.
partlist.xlsx	This file contains information of all the components used in the mechanical subsystem.

### Electronic subsystem

**Figure 1:**
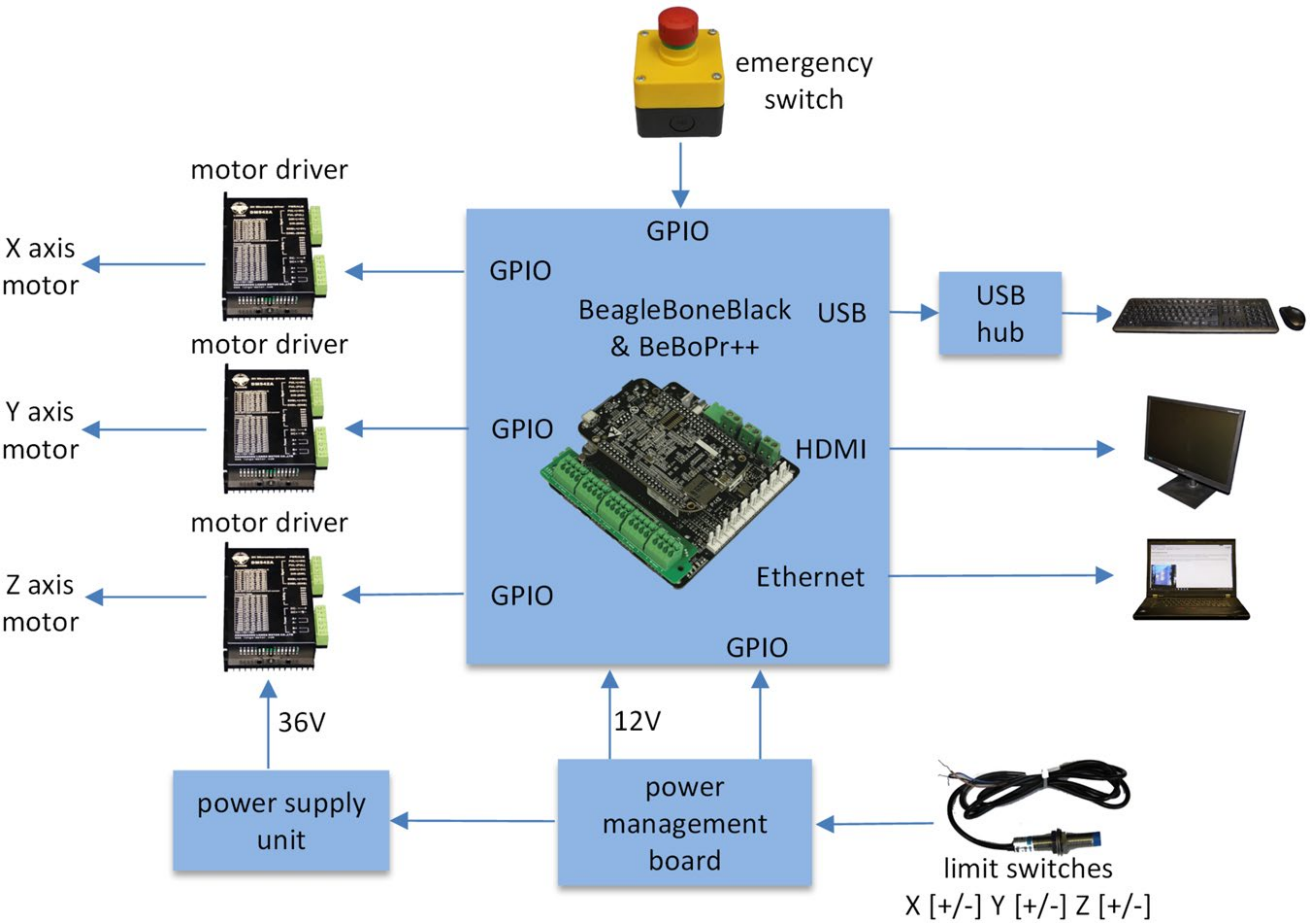
Schematic of the electronic subsystem. 3 stepper motors are controlled by Beagle Bone Black (BBB) through the BeBoPr++ base board and the motor drivers. 6 limit switches are connected to the BBB through level shifters on the power management board. An emergency switch is connected for safety reasons. The power supply unit and the power management board are in charge of generating, distributing and managing the power for the system. Peripherals such as monitor, keyboard and mouse are also connected.

**Figure 2:**
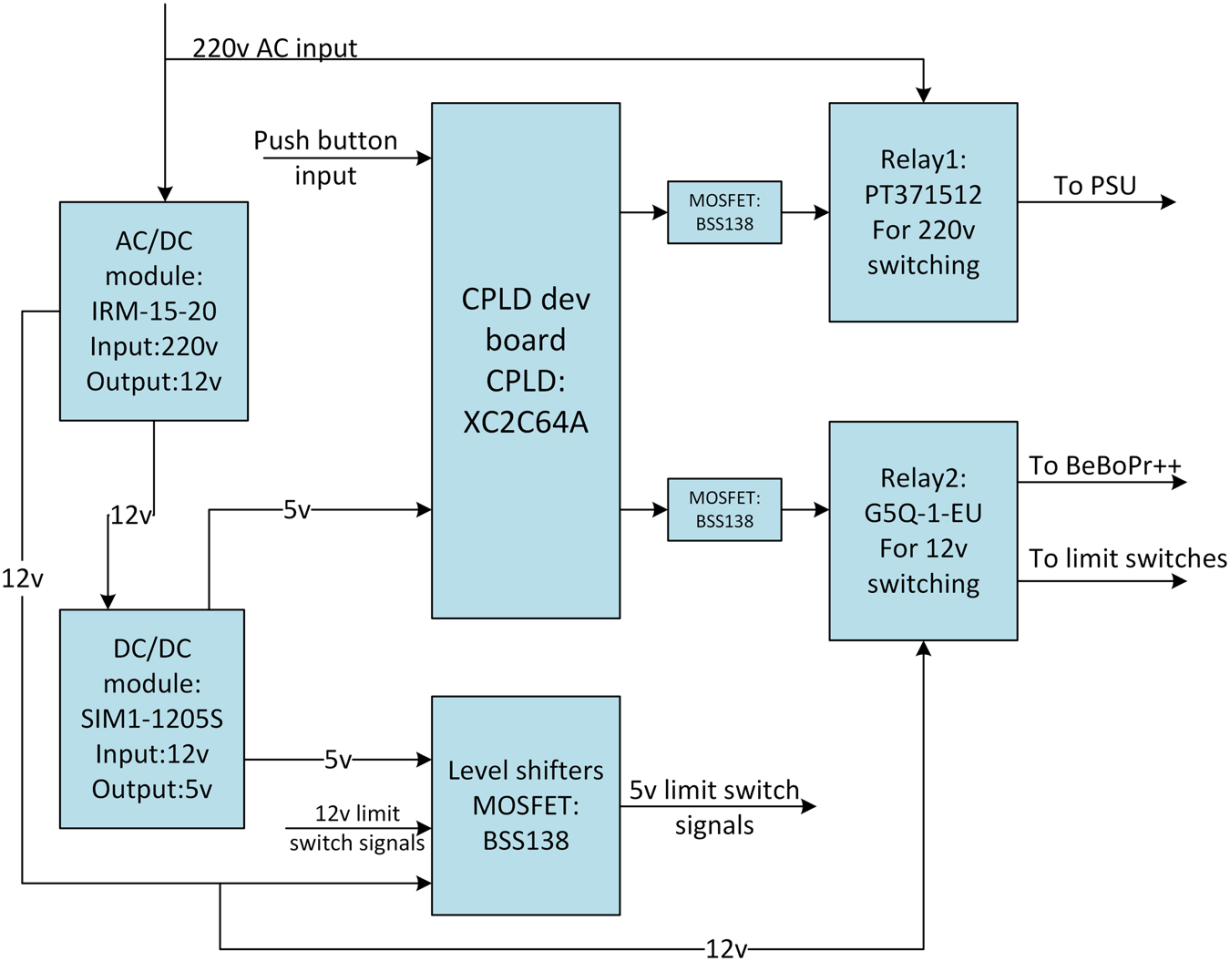
Block diagram of the power management board. The AC/DC module is used to generate the 12 V DC power for BeBoPr++/ BBB, level shifters and limit switches. The DC/DC circuit converts 12 V input to 5 V for level shifters and the CPLD. Two push buttons connected to the CPLD are used for controlling the power supply for motors and BeBoPr++/BBB separately by manipulating the relays.

The electronic subsystem consists of a community-supported open source embedded computer Beagle Bone Black (BBB)^53^, a cape board BeBoPr++ (http://elinux.org/BeBoPr%2B%2B), a power management board, a power supply unit (PSU), NEMA23 stepper motors, DM542A motor drivers and LJ12A3-4-Z/BX inductive proximity sensors. The schematic of the functionality is displayed in Fig. 1. The Beagle Bone Black is the heart of the electronic subsystem. It is equipped with an AM3358 (Texas Instruments, Dallas, TX, USA) which is a 1 GHz ARM Cortex-A8 System-on-Chip processor, 512MB DDR3 SDRAM (double data rate type three synchronous dynamic random-access memory) and versatile input/output options. The AM3358 is particularly suitable for real-time tasks like controlling motors since it contains two 32-bit microcontrollers which form the programmable real-time unit subsystem and industrial communication subsystem (PRU-ICSS). The BBB has 65 GPIO (general-purpose input/output) pins allowing for flexible application. The cape board BeBoPr ++ is designed to work with the BBB, which can be mounted on top. It is equipped with interfaces to connect up to 5 motor drivers, 6 limit switches, thermos sensors and PWM (pulse-width modulation) outputs for a possible CNC/3D-printing application. The BeBoPr++ also provides power (5 V direct current (DC)) for the BBB. All the signals interfacing BBB are protected on the BeBoPr++ so that the BBB would not be damaged. An emergency stop button was included for hardware protection and safety reasons. A power management board (Fig. 2 shows its block diagram) was designed with the open source EDA (electronic design automation) tool KiCad (version 4.0.2, kicad-pcb.org) to distribute the power in *COSI Measure* and to provide logic level conversion for the inductive proximity sensors. AC (alternating current) power is distributed to the PSU for the motors through a relay on this board. There is an AC/DC module on the board to generate 12 V DC power supply for the BeBoPr++. Two push buttons connected to a complex programmable logic device (CPLD: XC2C64A, Xilinx, San Jose, CA, USA) are used to control the AC and DC power on and off independently allowing turning the BBB on and leaving the motors off in order to save energy. Delay circuits are implemented in the CPLD so that a short press on the buttons turns the power on while a long press turns the power off. Six inductive proximity sensors with a detection distance of 4 mm were used as limit switches to detect the working range of *COSI Measure*. These sensors require a minimum working voltage of 6 V DC which is higher than the interface logic (5 V transistor–transistor logic (TTL)) on the BeBoPr++. Hence six logic shifters were implemented on the power management board. We used three NEMA23 stepper motors (3 A@36 V, 3NM holding torque, 1.8° step angle) together with three DM542A motor drivers, which allow 2-128x micro-stepping leading to a theoretical precision limit of ~200 nm and a half holding current operation for power saving. We chose to use stepper motors mainly for three reasons: 1) Stepper motors provide a submillimetre resolution. 2) They are easy to operate and to deploy. Stepper motors have positional control via its nature of rotation by fractional increments, so that no feedback (closed-loop) is needed for accurate positioning. 3) Stepper motors are less expensive than alternatives such as servo motors or piezoelectric/ultrasound motors. The motors and drivers are supplied by an AC/DC power supply unit (36 V, 9.7 A). The electronic components have been integrated into a PC (personal computer) chassis for easy transportation. An HDMI (high-definition multimedia interface) monitor, a keyboard, a mouse as well as an RJ45 Ethernet cable were connected to the BBB via the chassis, so they form a complete standalone computing system. Cables from the stepper motors and limit switches were inserted into cable chains and soldered to miniature circular connectors. This allows flexibility in removing the standalone *COSI Measure* PC to be unplugged and used for other applications.

PCB (printed circuit board) design files of the power management board (schematic & layout), bill of materials, connection guide and the CPLD source code are available in the files provided, with an overview given in Table 2.

**Table 2:** Design files for the electronics subsystem.

File/folder	Contents
BOM.xlsx	This file contains information of all the components used in the electronic subsystem.
COSI_pwr_board	This folder contains the project files of the power management board, e.g. schematic and layout files.
COSI_pwr_CPLD	This folder contains the project files for the CPLD on the power management board.

### Software

**Figure 3:**
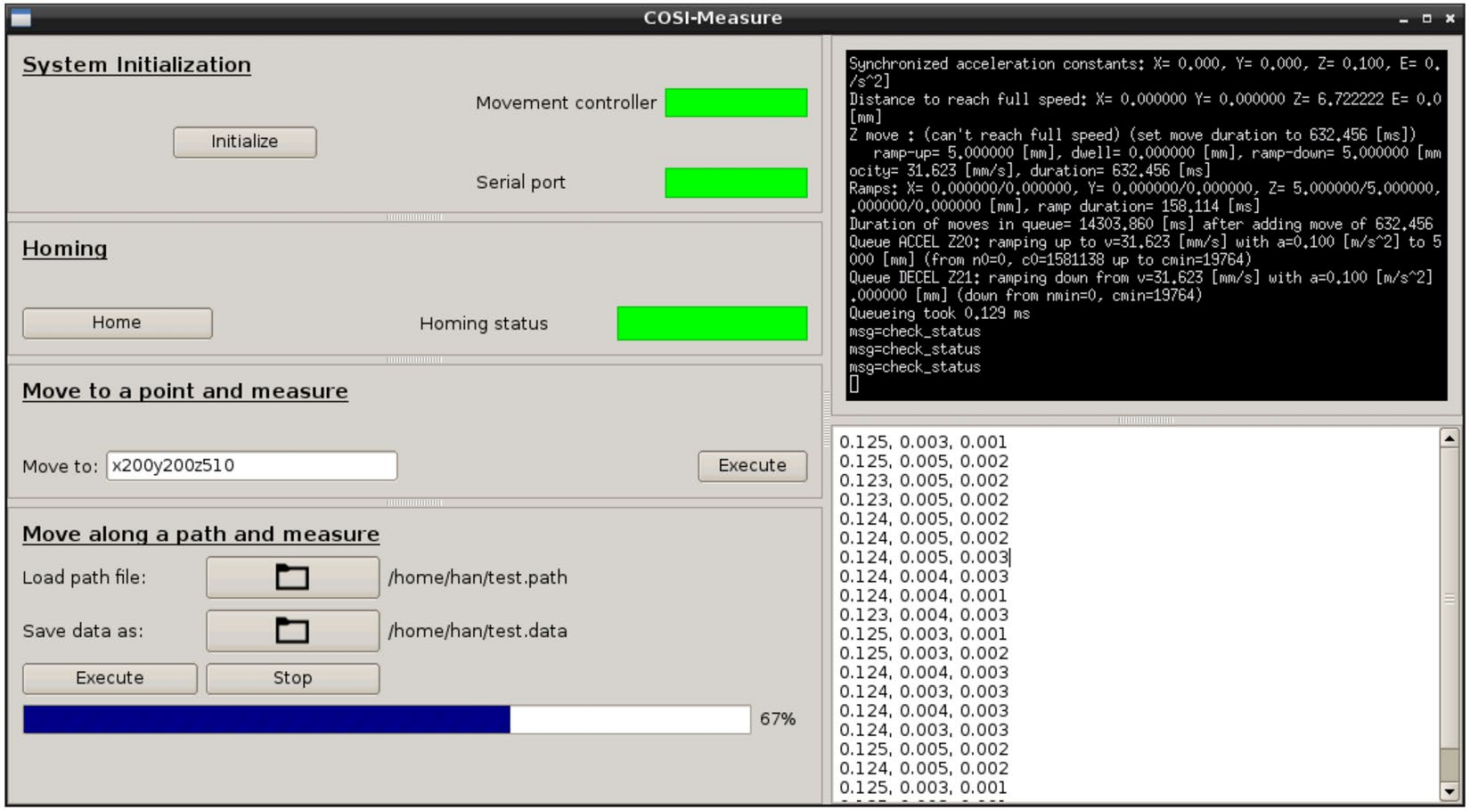
Graphical user interface for COSI Measure. The left side of the GUI contains windows of the four functions: system initialization, homing, single coordinate measurement and 2D/3D path measurement. The top half of the right side of the GUI displays the status of the motor movements while the bottom half shows the measurement readouts.

The latest firmware and instructions for the installation of the BBB can be found at http://beagleboard.org/latest-images. We are running Debian 7.11. The motor controller program contains two parts: PRU code and ARM code. The PRU code comes in the form of a binary module with the BeBoPr++ and is proprietary. Yet, the PRU code is stored in the memory chip on the BeBoPr++. It is shipped together with the BeBoPr ++, so any lab that follows our part list and instructions will have the PRU code at no additional cost. The PRU code runs on the co-processors and controls the timing and acceleration for the stepper motors. The ARM code is open source and well commented. It forms a layer above the PRU code and is responsible for tasks like command parsing, G-code (MIT, Cambridge, MA, USA) processing, distance calculation, etc. This software works with command line interface and responds to G-code commands by user input. We forked the project from https://github.com/modmaker/BeBoPr and adapted it for *COSI Measure*. A graphical user interface (GUI, Fig. 3) was developed with PyQt (version 4.9.3)^54^ to integrate all the functions, so that we could have a more user friendly interface than the command line interface. It provides four functions:

1. System initialization: Initialize the motor controller and the serial port, which connects to the measuring instrument (e.g. a Gaussmeter). A server was implemented in the motor controller ARM code. During system initialization the GUI starts the motor controller and establishes a link to the server so that the GUI can send commands to the motor controller and check its status. Two status indicators represent the status of the motor controller and the serial port respectively. A green indicator indicates the motor controller or the serial port functioning correctly, otherwise red. A sub-window shows the motor controller interface.
2. Homing: Move along the whole measurement range until x_min_, y_min_, z_min_ and x_max_, y_max_ and z_max_ are found. An indicator will turn green when this process is finished without error.
3. Single coordinate measurement: Move to a single point and read sensor data. The machine moves to a location specified by the user (coordinates are in millimeters, e.g. × 100y200z300) and then takes a measurement. The readout is then displayed on a sub-window of the GUI.
4. 2D/3D path measurement: Move and measure along a user defined trajectory. The user should provide two files in which one contains the trajectory (each line contains one coordinate in millimeters); the other will have the raw measurement results appended. A progress bar was implemented to show the measuring process.

The software project can be found under “software” directory in the project repository.

### Measurements

*COSI Measure* was evaluated for backlash, precision, speed and strength. In order to investigate the precision of the system and the reproducibility of the position after several iterations, a home-built 1 W 405 nm laser device attached to the probe holder was used to engrave dots on a piece of paper. The experiment setup and engraving process was recorded in Supplementary Video S1. For testing the precision of *COSI Measure*, the laser device was first moved along a trajectory with defined coordinate points (41 points along a straight line 0.5 mm apart) and with a 0.2 s stop at each point to engrave the paper. The laser device was then moved 10 iterations following the same pattern with a homing process inserted after the fifth iteration to fully test the backlash/reproducibility performance of the system. A series of concentric circles (7 circles with 1 mm radius increments starting from 1 mm, each circle has 12 evenly distributed points) were also programmed to further demonstrate the precision of the system. This experiment was repeated on x-y plane, x-z plane and y-z plane respectively for a thorough 3D evaluation. A digital calliper (resolution = 0.01 mm, Orion Tool, IL, USA), a ruler (resolution = 0.5 mm, Orion Tool, IL, USA) and a camera (EOS60D, Canon, Tokyo, Japan) were used to measure the distance between the engraved dots.

**Figure 4:**
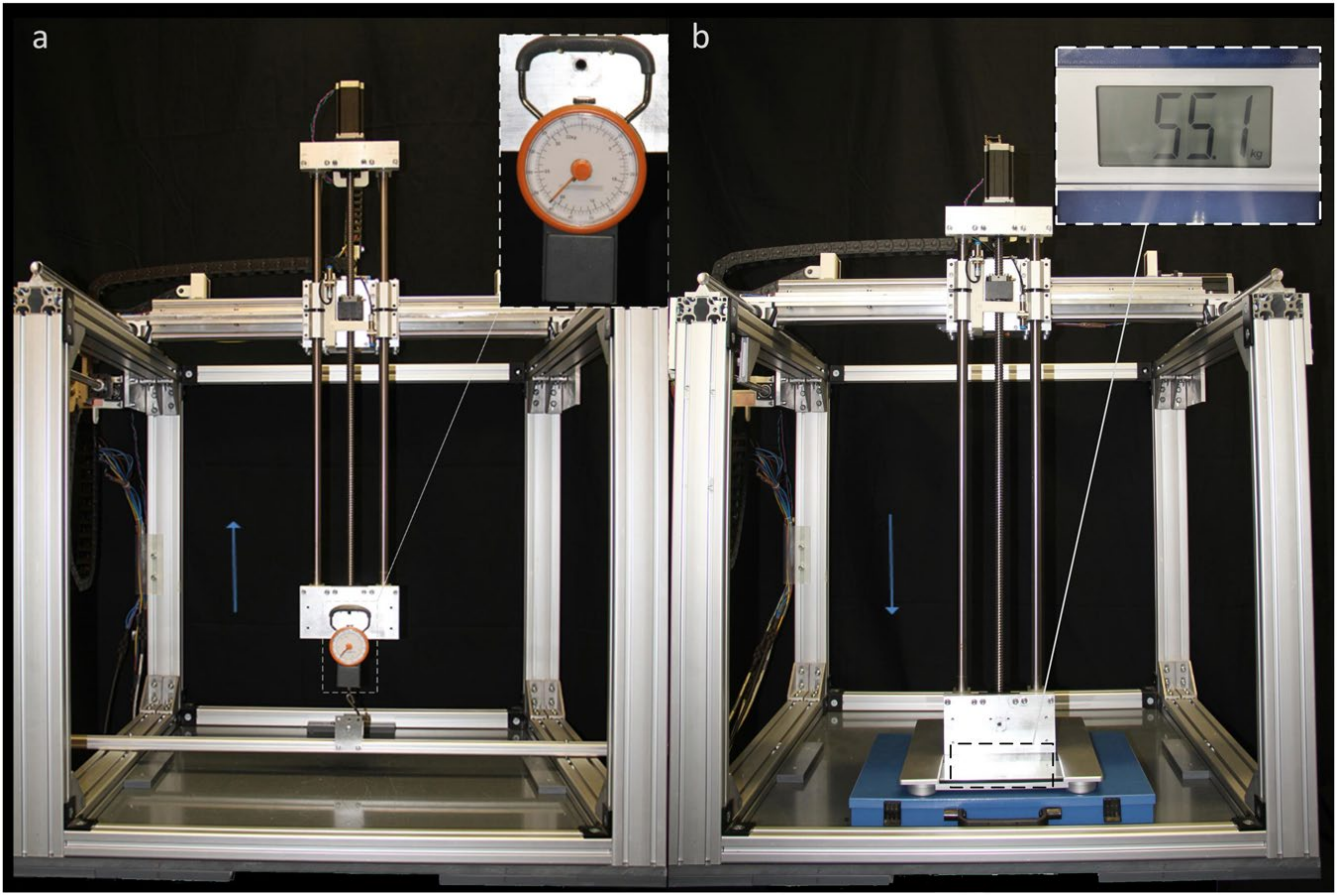
Experiment setup for testing force and load. (**a**) A force meter was attached to the probe holder and hooked to the frame to test the maximum load. It shows 21 kg when COSI Measure cannot move further. (**b**) A scale was put under the probe holder to test the maximum force. It is 55.1 kg when COSI Measure is unconstrained (not fixed to the ground).

In order to measure the maximum load of the machine, a force meter (Samsonite, Luxembourg City, Luxembourg) was attached to the probe holder and hooked to the frame (Fig. 4a). *COSI Measure* was then moved vertically along the z-axis upwards. By reading the force meter at the point when *COSI Measure* could no longer move further, we could get its maximum load. A scale (Model: Pharo, Leifheit AG, Nassau, Lahn, Germany) was put under the probe holder (Fig. 4b) to test the maximum force. Similarly, we made the machine move vertically along the z-axis downwards; from the scale we can get the maximum force information. This parameter was examined in two scenarios: constrained (*COSI Measure* was fixed to the ground) and unconstrained. We also measured the time taken for the system to move along a single axis over a distance of 500 mm in order to get the speed of the system. Each measurement (load, force and speed) was repeated for 10 times to get an average result.

To demonstrate high fidelity automatic field mapping in a scientific application, a Gaussmeter (Model 460, Lake Shore Cryotronics, OH, USA) probe was positioned in a customized 3D printed probe holder (CAD files available) to map the magnetic field of a permanent magnet in a Halbach ring arrangement which will be used in the construction of a prototype magnet for an open source MRI scanner^19^. The experiment was partly recorded in Supplementary Video S2. The field mapping results were compared to magnetostatic field simulations (CST MWS 2015, Darmstadt, Germany) performed with the same Halbach ring configuration. *COSI Measure* moves along a 2D spiral trajectory (inside to outside) defined by an input path definition file. A slice in space with an area of (60 × 60) mm^2^ and a spatial resolution of 2 mm was covered in the mapping. After each coordinate *COSI Measure* interacts with the Gaussmeter and records the measured values of the 3-axis Hallprobe (MMZ-2508-UH, Lake Shore Cryotronics, OH, USA).

Stray magnetic field measurements were conducted for an actively shielded 3.0 T (Magnetom Verio, Siemens Medical, Erlangen, Germany) and a passively shielded 7.0 T (Magnetom, Siemens Medical, Erlangen, Germany) whole body MR scanner. Supplementary Video S3 depicts sequences of the measurement of the 3.0 T MR scanner. The magnetic field was measured with a 3-axis Hallprobe (MMZ-2508-UH, Lake Shore Cryotronics, OH, USA) in two (50 × 50) cm^2^ transverse planes at a 90 cm distance to the MRI scanner. The centre of the planes was aligned with the horizontal centre line of the MR scanner bore. With a spatial resolution of 5 mm, 10201 (101 × 101) points were acquired in total for each plane. The Hallprobe was attached to an aluminium rod (length = 129 cm for 3.0 T, length = 255 cm for 7.0 T) which was attached to the probe holder of *COSI Measure*. A 3 seconds pause was programmed after each movement to reduce the vibration at the tip of the extension rod.

At last, the temperature distribution of an RF heating coil was examined. This shielded eight-rung highpass birdcage coil with an inner diameter of 20 cm was designed for scrutinizing RF power deposition induced heating^38^. A phantom (8 × 9 × 8 cm^3^) containing 650 ml fumed silica and water mixture (1 g: 5 g) was placed in the centre of the RF coil as an object under (heating) investigation. A signal generator (Model: SMGL, Rohde & Schwarz, Munich, Germany) and a power amplifier were used to drive the coil with an average output power of ~70 W at 297 MHz. A power meter (Model: NRT, Rohde & Schwarz, Munich, Germany) was inserted between the amplifier and the coil to monitor the power output. A fibre optic temperature sensor (Model: TC, Neoptix Inc, Québec City, Québec, Canada) attached to the probe holder of *COSI Measure* was used to capture the temperature profiles across the phantom after reaching the heating equilibrium (about 120 min). A fibre optic temperature monitor (OmniFlex, Neoptix Inc, Québec City, Québec, Canada) was connected to the sensor to log temperature data. The same RF power was applied to the RF coil during the mapping process to maintain the equilibrium. A (50 × 50) mm^2^ horizontal slice aligned with the centre of the RF coil was selected with a 1 mm spatial resolution. The mechanical subsystem of *COSI Measure*, the RF coil and the phantom were fixed inside the 7.0 T MR scanner room to take advantage of the shielding of the scanner room, so that the experiment would not generate electromagnetic interferences. Supplementary Video S4 illustrates the temperature measurement.

### Data availability

The data that support the findings of this study are available from www.opensourceimaging.org.

## Results

**Figure 5:**
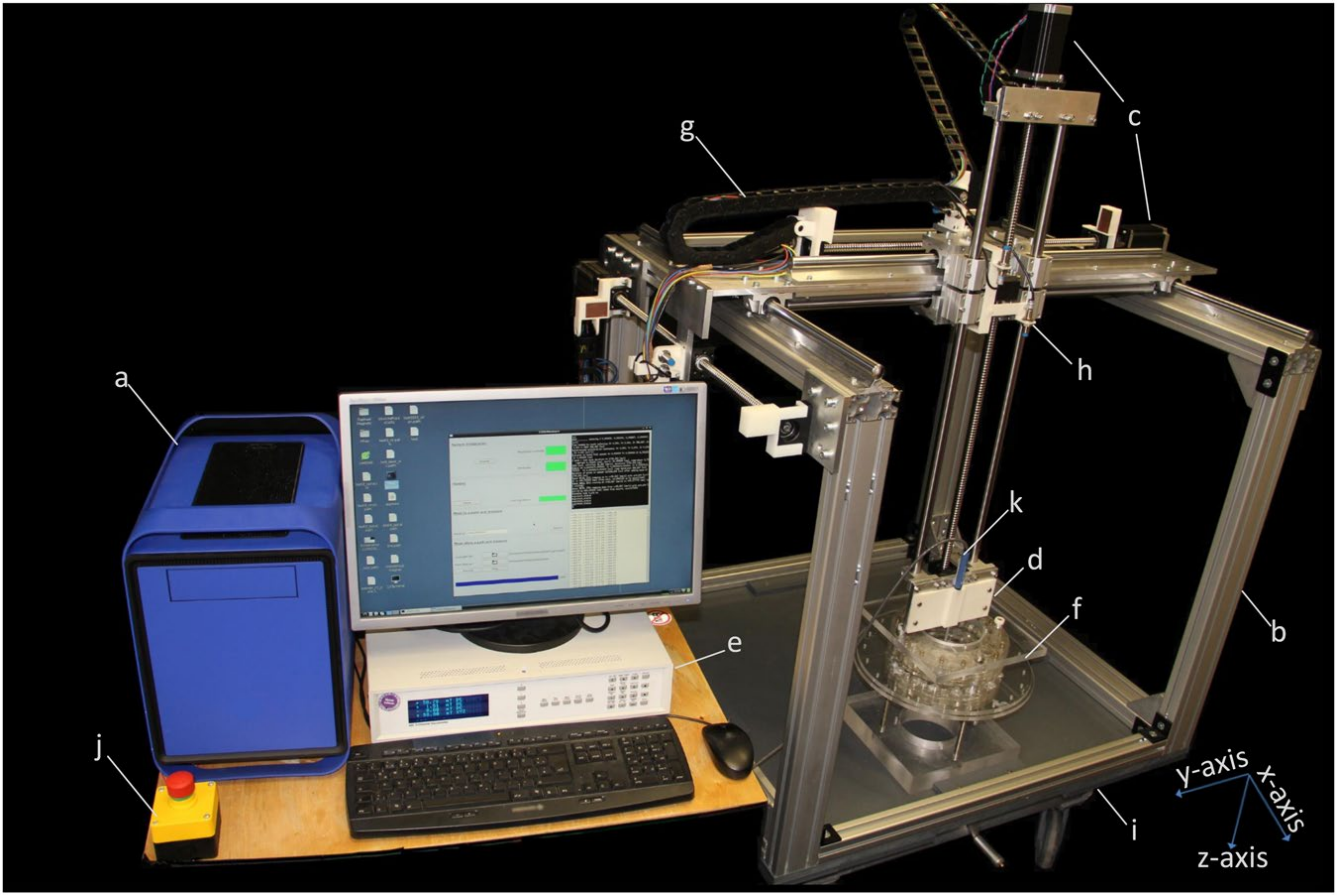
COSI Measure, a low cost precise open source multipurpose measurement system. (**a**) personal computer chassis with electronic components inside (**b**) aluminium frame (**c**) motors (**d**) probe holder (**e**) Gaussmeter (**f**) Halbach ring magnets as object under investigation (**g**) cable chain holder (**h**) limit switch (**i**) movable lift table (**j**) emergency push button (**k**) Hall sensor probe ((**e**), (**f**) and (**k**) are not part of COSI Measure, they are shown here for illustrating the measurement setup.)

Figure 5 demonstrates the *COSI Measure* system measuring a home-built Halbach magnet. The maximum working volume of *COSI Measure* is (530 × 530 × 640) mm^3^ with a maximum moving speed of 37 mm/s. The maximum load on the probe holder is 21 kg which makes the machine capable of supporting heavy scientific instruments and tools. While it is not fixed to the ground, the maximum force which the machine can apply to an object vertically downwards is 540.35 N (55.1 kg). This parameter increases to 896.33 N (91.4 kg) when *COSI Measure* is fixed to the ground.

**Figure 6:**
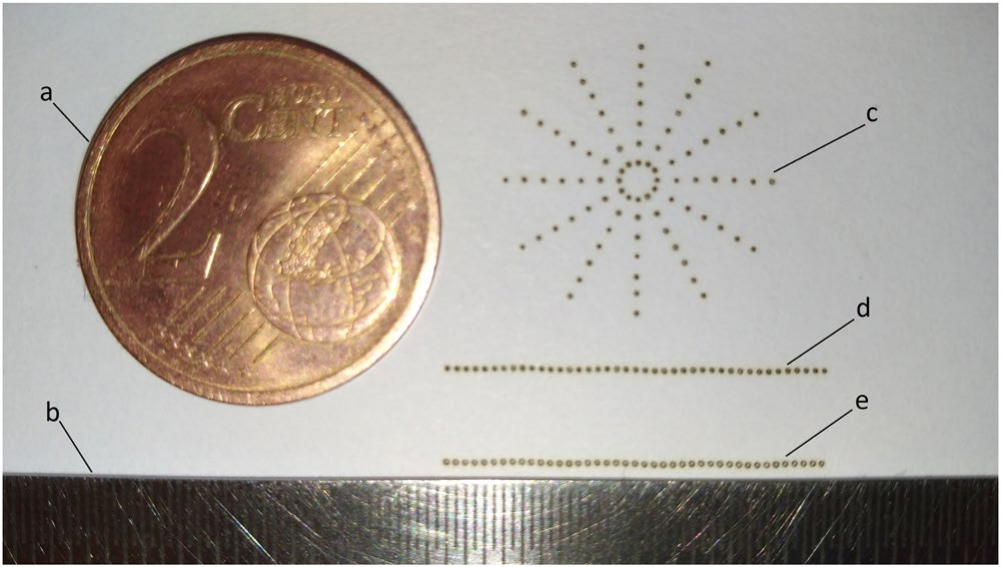
Precision and reproducibility results. (**a**) a 2 cent Euro coin, diameter 18.75mm (**b**) the ruler, resolution = 0.5 mm (**c**) 7 concentric circles with 1 mm radius increments starting from 1 mm, each circle has 12 evenly distributed points (**d**) a straight line contains 41 points with 0.5 mm apart, engraved in one iteration (**d**) a straight line contains 41 points with 0.5 mm apart, engraved in ten iterations.

First, the spatial fidelity and reproducibility of *COSI Measure* was evaluated by laser engraving a computer defined dot pattern consisting of 41 points along a straight line 0.5 mm apart. Figure 6 illustrates the results of the precision and reproducibility measurements for the x-y plane. The upper line was engraved in one iteration along the y-axis and the lower line was burned into the paper in ten iterations including a homing process inserted after the fifth iteration. The average diameter of the points increased from 0.1 mm to 0.25 mm when they were engraved multiple times. The laser device returned to exactly the same location at the first point after each iteration. Even with the homing process inserted, no visible backlash was observed. A maximum deviation of 0.16 mm on the x-axis (perpendicular to the moving direction y-axis) was measured. Table 3 contains the detailed measurement results for the distance between two adjacent points for the experiment on x-y plane. The 7 concentric circles were in full accordance with the programmed trajectory definition and showed ample precision of the system with 2D movements. Experiments on x-z and y-z plane exhibited almost identical results. These results demonstrate the submillimetre precision and underline the reproducibility and backlash free performance of *COSI Measure*.

**Table 3:** Measurement results for the distance between two points on x-y plane (mm).

	Mean	Minimum	Maximum	Standard deviation
1 iteration	0.50	0.47	0.52	0.08
10 iterations	0.50	0.48	0.53	0.07

**Figure 7:**
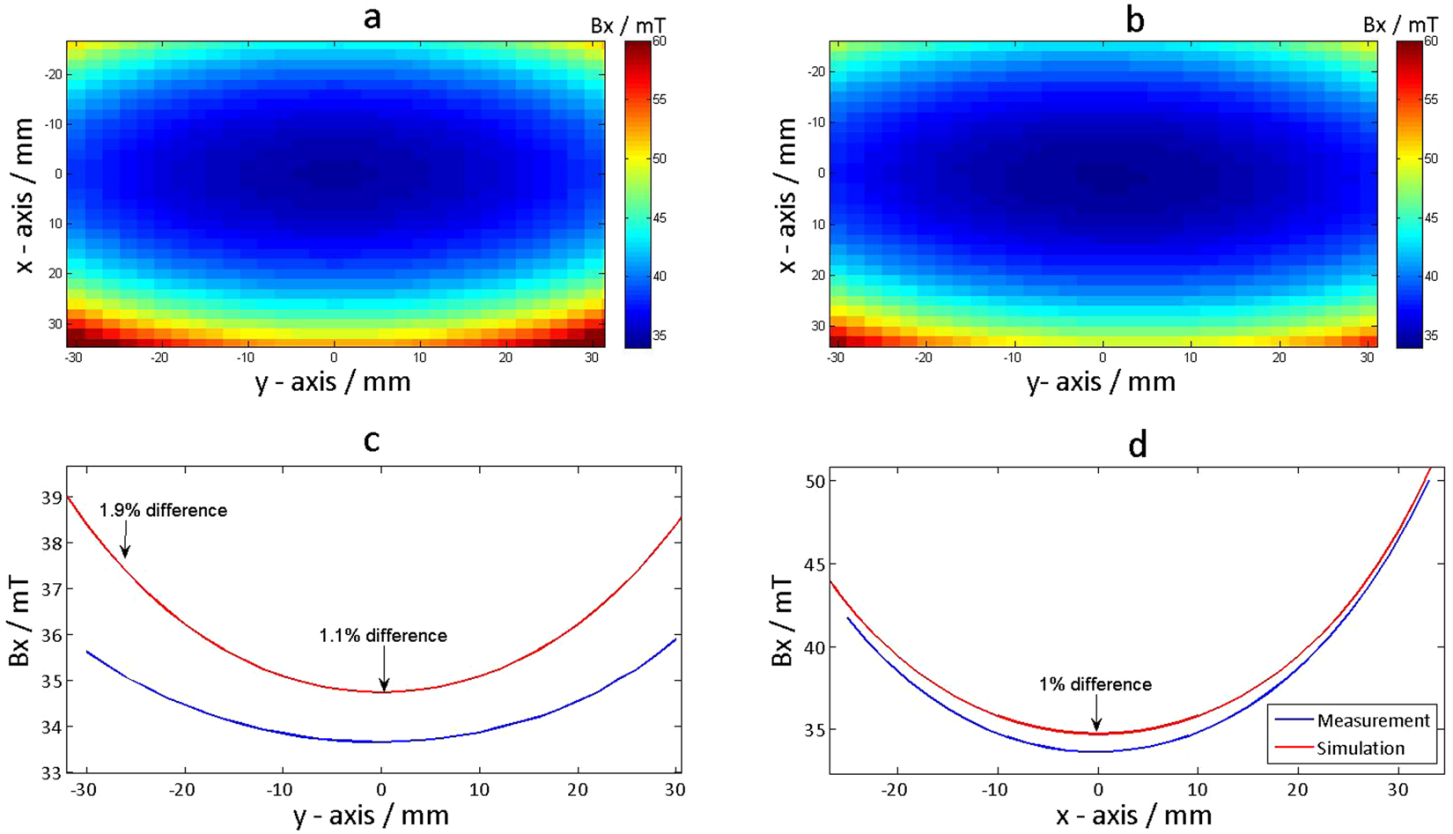
Magnetic flux density mapping results of a Halbach ring magnet compared with simulation results. (**a**) Measurement results of a 60 × 60 mm^2^ slice with the centre of the ring being the isocenter, the spatial mapping resolution was 2 mm. (**b**) Simulation results with the same setup. (**c**) Comparison of the line plots through the isocenter between the measurement and simulation on the y-axis. A maximum deviation of 1.9% was observed. (**d**) Comparison between of the line plots through the isocenter the measurement and simulation on the x-axis. A maximum deviation of 1% was observed.

Next we replaced the laser device by an H-field sensor to examine the applicability of *COSI Measure* for the assessment of the magnetic field distribution of a single ring Halbach magnet with a diameter of 13.85 cm. These measurements are essential to benchmark the field uniformity against the field distribution deduced from magnetostatic field simulations which were undertaken during the design phase of the development process. Figure 7a shows the measured in-plane magnetic flux density distribution of the Halbach magnet. For comparison the field dispersion deduced from the magnetostatic field simulation of the same Halbach magnet design is depicted in Fig. 7b. Figure 7c,d highlight the comparison between the measurements and simulation results for the same magnet through the magnet centre along y- and x-axis respectively. A maximum deviation of 1.9% was observed between simulated and measured magnetic flux density, which provides major encouragement for further development, simulation and implementation of novel Halbach magnet designs.

**Figure 8:**
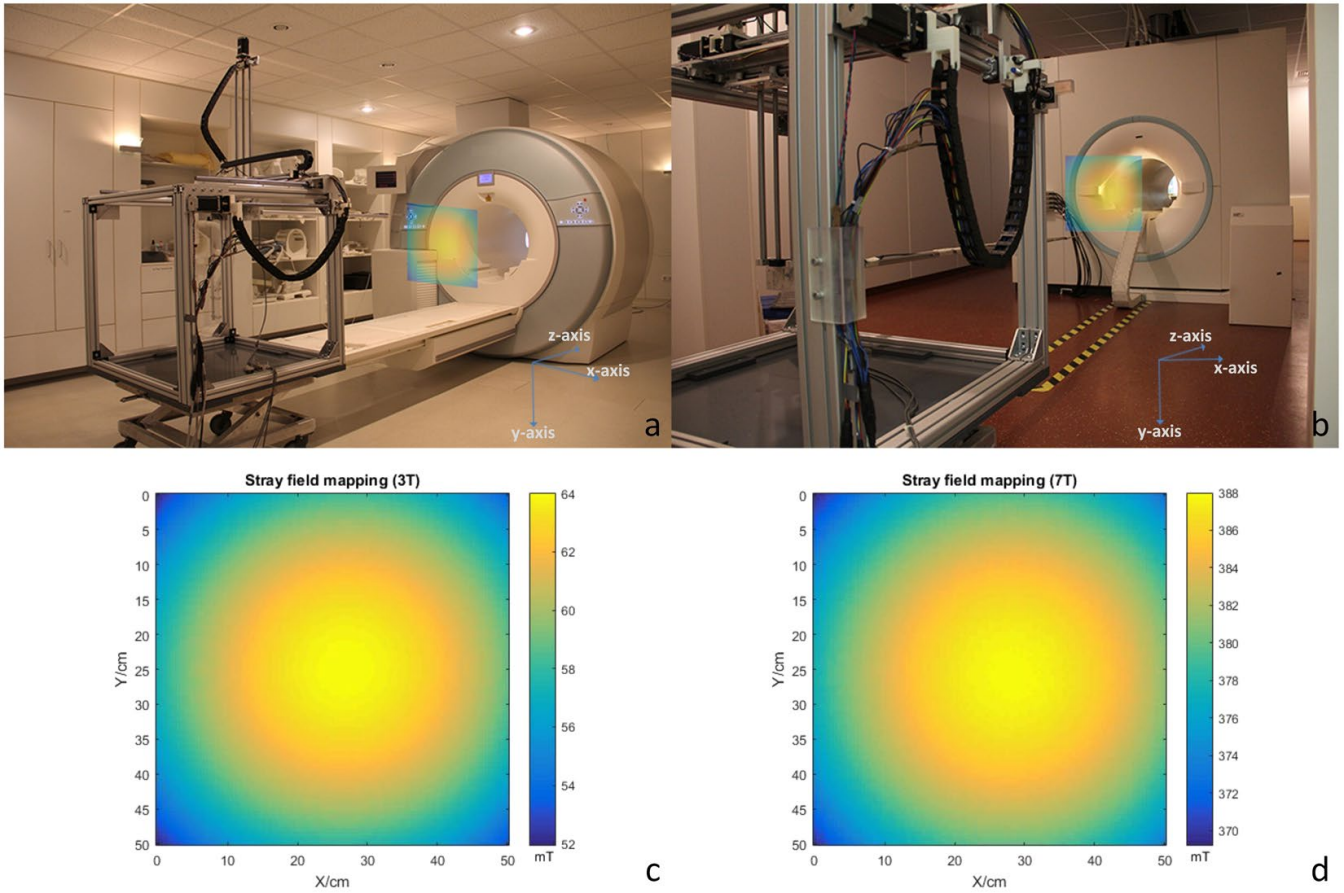
Stray field mapping setup and results. (**a**) Stray field mapping setup in the 3 T scanner room, a 50 × 50 cm square transverse slice 90 cm outside the bore was selected. (**b**) Stray field mapping setup in the 7 T scanner room, a 50 × 50 cm square transverse slice 90 cm outside the bore (from the back side of the scanner) was selected. (**c**) Stray field mapping results (vector magnitude) of the selected slice for the 3 T scanner (spatial resolution: 5 mm). (**d**) Stray field mapping results (vector magnitude) of the selected slice for the 7 T scanner (spatial resolution: 5 mm).

Next we extended the probe head by an aluminium rod and attached an H-field sensor horizontally to the tip of the rod. *COSI Measure* was positioned at the rear end of the patient table of a 3.0 T MR scanner and at the back side of a 7.0 T MR scanner to characterize the stray magnetic field strength and uniformity in an area close but aligned to the magnet bore. We consider this stray field to be used for MRI at low magnetic field strengths similar to that of the Halbach magnet. The vector magnitude derived from the stray field mapping of the 3.0 T and 7.0 T MRI scanners are summarized in Fig. 8c and d. Positioning *COSI Measure* at the end of the patient table of the actively shielded 3.0 T system with a 129 cm rod supporting the H-field sensor did not affect its functionality and did not evoke too excessive attraction forces due to the stray field of the MRI system, which is approximately 2.5 mT at this position. For the passively shielded 7.0 T magnet the inductive limit switches started to malfunction at a distance of ~3 m away from the magnets rear end where the stray field was around 45 mT. Notwithstanding this minor malfunction the stray field could be successfully mapped using a longer aluminium rod with a length of 255 cm to accommodate and guide the H-field sensor. The measured fields for both the 3.0 T and the 7.0 T magnet show an expected maximum B_0_ in the centre of a plane perpendicular to the B_z_ direction. The smooth transitions in the field plot demonstrate that minor vibrations of the base system, which might be amplified over the length of the rod are small enough (<5 mm mapping resolution) to not disturb the measured data (Fig. 8c and d). Our measurements underline the excellent field uniformity of the stray field and make this stray field an ideal candidate for MRI in a low field environment of a commercial superconducting magnet to be used as a reference for our results obtained from a home-built low field permanent Halbach magnet.

**Figure 9:**
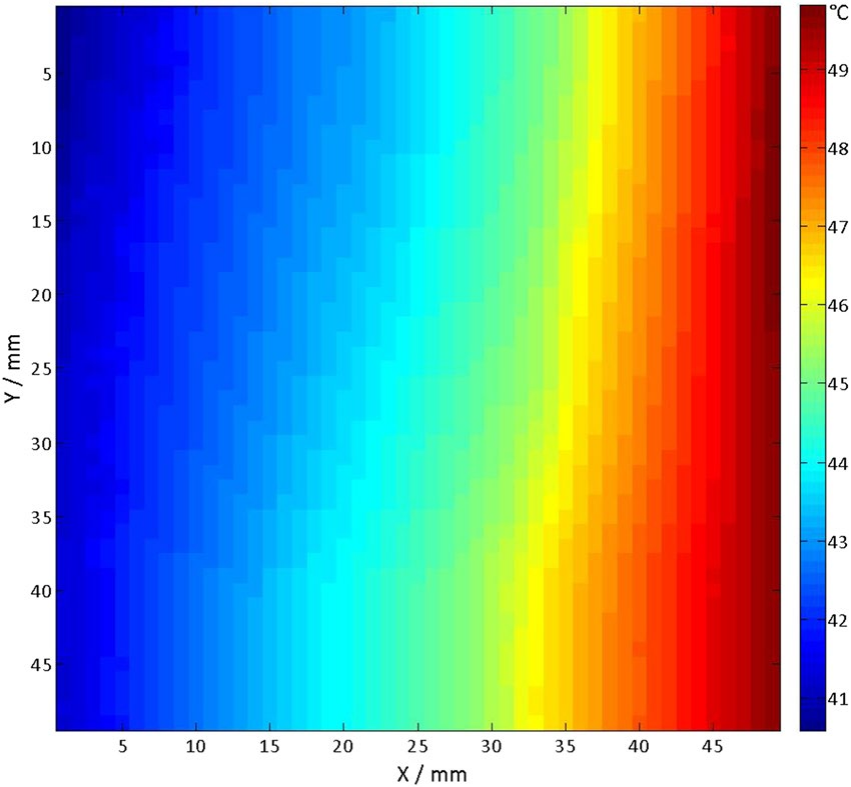
2D fibre optic temperature mapping result. Temperature mapping result after reaching the RF heating equilibrium of the silica/water phantom. A (50 × 50) mm^2^ horizontal slice aligned with the centre of the RF coil was selected and sampled with a 1 mm spatial resolution. Initially the phantom had a temperature of 21.9 °C.

To take *COSI Measure* to the next application, a fibre optic temperature sensor was attached to the probe holder to capture the temperature profile across a phantom placed in the RF coil used for RF induced heating. The temperature distribution of the heated phantom is illustrated in Fig. 9. This 50 × 50 mm^2^ temperature mapping has a spatial resolution of 1 mm. The map was acquired in ~100 min (2.4 s per point) right after the thermal equilibrium was achieved, which took 120 min with 70 W signal driving the coil and an initial phantom temperature of 21.9 °C. At the right side of the phantom, which is closer to the feeding point of the RF coil, higher temperatures are observed. The result indicates that *COSI Measure* is able to move smoothly not disturbing the temperature distribution in the object under investigation.

## Discussion

An automated open source and versatile 3D measurement system (*COSI Measure*) was developed and validated. Our main findings are that *COSI Measure* supports a submillimetre spatial resolution that can be utilized for a broad range of measurements in MRI and related fields.

For the system design we ensured that all materials are easily accessible by the community. For example, the motors, proximity sensors and aluminium frames are standardized components which are made readily available by numerous vendors. We customized *COSI Measure* in a way that it can be built and assembled conveniently. For example, instead of soldering a 44-pin CPLD chip onto the power management board, we chose to use a small and cheap CPLD development board plugged onto the power management board. Our goal was to make *COSI Measure* affordable. For example, we employed inexpensive yet accurate enough stepper motors and inductive proximity sensors. It is a strength of *COSI Measure* that it employs open source hardware and software whenever possible. The Beagle Bone Black is an open source design. The operating system (Linux) and the ARM motor control program are also open source. To ensure reproducibility of *COSI Measure* and our proof-of-principle experiments we provide a detailed part list, in-depth assembly instructions and detailed documentation of our experiments.

*COSI Measure* consists of 3-orthogonal axes with a working volume of (530 × 530 × 640) mm^3^. It is open to all dimensions allowing for flexible measurement positioning. The experimental results demonstrated submillimetre spatial fidelity and reproducibility of the system and no significant backlash. *COSI Measure* is able to carry a load of up to 21 kg, which supports the attachment of a broad range of probe heads including temperature, ultrasound, optical, radiation and electromagnetic sensors and probe holders.

By equipping *COSI Measure* with a Halbach probe the capability for mapping magnetic fields was demonstrated using a Halbach magnet as an example. Our measurements detected an absolute deviation between magnetostatic field simulations and measurements of only 1.9%. In another example we illustrated the performance and utility of *COSI Measure* for mapping the stray magnetic field of an actively shielded clinical 3.0 T MRI scanner and of a passively shielded human 7.0 T MRI scanner. The strong magnetic stray field of the passively shielded 7.0 T set the limit of a distance of 3 m (~45 mT) for *COSI Measure*. At this field strength the inductive limit switches started to work outside of their specification. Changing the limit switches to mechanical configurations would address this malfunction. However, when positioning the base of *COSI Measure* into stronger magnetic fields magnetic forces of the magnet on the stepper motors become too large. This limitation can be relaxed by using extensions of the probe head. A rigid extension might magnify the vibration of the system but will not hamper the fidelity of *COSI Measure*. With an extended rigid probe holder in-bore measurements are feasible for 7.0 T, 3.0 T magnets and at lower magnetic field strengths. *COSI Measure* would be even capable to support data sampling in the isocenter of a 9.4 T human MR scanner as long as a proper probe holder is provided. Arguably, extended probe heads increase the sensitivity of the measurement system to vibrations, which need to be accounted for by using decreased speed and/or longer waiting times for each measurement point. Using a probe holder with a length of 255 cm allowed successful mapping of the magnetic field in a plane located at a 90 cm distance from the rear end of a 7.0 T MR magnet. For this setup no extra infidelity was caused by vibration due to the use of a 3 s waiting time per measurement point.

*COSI Measure* is well suited for conducting RF antenna/coil characterization experiments by mapping the E-field, the H-field or the temperature as demonstrated by the temperature mapping results. This is of particular interest for explorations into thermal MR^35–37^ and MR safety^38–41,44^ where the measurements of RF induced temperatures might require submillimetre resolution. *COSI Measure* enables such measurements which are essential for designing new RF transmission antennae, for validating findings obtained from electromagnetic field simulations or for investigations into RF induced heating of electrically conductive implants. Admittedly, covering a 50 × 50 mm^2^ area for temperature mapping with a sampling rate of ~2.4 s per point consumed ~100 min in this study. This time can be reduced significantly for the experimental setup proposed here. Attaching an array of fibre optic sensors and reducing the sampling time per point would further accelerate data acquisition while maintaining the superb spatial resolution (1 mm).

The open-source nature, large working volume, robust configuration and high fidelity of *COSI Measure* make it not only suitable for advancing MR research but also for a variety of scenarios where accurate positioning and repeated movements are necessary. In millimetre-wave communication/radar systems, where antenna characteristics such as radiation pattern, reflection and scattering are important, a 3D test setup for characterization is usually required^55–59^. With minor modifications (e.g. adding an absorption cover), which can be conveniently accomplished since all the technical details of *COSI Measure* are freely accessible, *COSI Measure* can be easily turned into a 3D antenna test bench. Another possible application could be in durability/fatigue testing which usually involves cyclic movements and is common in aerospace, automotive, defence, manufacturing and electronic industries. *COSI Measure* already provides a robust setup, with extra components added (e.g. a load cell); it can also be used as a fatigue testing system.

Further improvements of *COSI Measure* include automatic mapping of an object using distance sensors or a camera together with computer vision algorithms^60^. This would allow one to easily map the field of complex geometries such as RF coils, medical implants etc., which otherwise requires prior planning of the measurement path based on location and geometry of the object to measure. The moving speed of the machine could also be improved by further tuning the motor control algorithm. The observed maximum 0.16 mm deviation on the axis perpendicular to the moving direction was induced by the imperfection of the threaded shafts in the ball screws. This can be improved by replacing the shafts with more precise ones.

Parallel to the publication of this manuscript soft- and hardware of *COSI Measure* will be made available open source on www.opensourceimaging.org. Open source provides unique opportunities to modify and expand the capabilities of *COSI Measure* for a broader range of applications. With a downward force of >500 N (50 kg) mechanic tools such as a drill, saw or mill can be mounted to the probe holder. The submillimetre precision of the base system also affords 3D printing or SMD soldering. These applications are already supported by the hardware and hence would require only minor modifications in software along with setting up a dedicated holder tailored for the particular tool or probe head. In a resource limited research setting, such an approach allows for an efficient use of laboratory space, financial resources (both investment and maintenance) and collaborative efforts of the research community to extend the functionality. To summarize *COSI Measure* is in full alignment with the idea of open access for everybody and with the needs of reproducibility and rigour of research as requested by numerous authorities, the various funding bodies, the general public and the research communities.

## Electronic supplementary material

Supplementary Video S1

Supplementary Video S2

Supplementary Video S3

Supplementary Video S4
